# The gut microbiome as an actionable drug-sensitivity modulator for immune checkpoint blockade: clinical evidence for FMT, live biotherapeutics, and defined consortia

**DOI:** 10.3389/fimmu.2026.1802676

**Published:** 2026-03-24

**Authors:** Wenqian Han, Quanfang Li, Guangwen Yuan

**Affiliations:** 1 Department of Pharmacy, The Affiliated Taian City Central Hospital of Qingdao University, Tai’an, China; 2 Department of Drug Clinical Trials, Zibo Central Hospital, Zibo, China

**Keywords:** fecal microbiota transplantation, gut microbiome, immune checkpoint inhibitors, live biotherapeutic products, microbiome engineering

## Abstract

Immune checkpoint inhibitors (ICIs) deliver durable benefit to only a subset of patients and can be limited by immune-related adverse events (irAEs). The gut microbiome has emerged as an actionable, host-level modulator of ICI drug sensitivity and toxicity. This mini-review links microbial ecology to antigen presentation, T-cell priming and fitness, metabolite signaling, and barrier inflammation, and summarizes interventional evidence across three modalities. Responder-derived fecal microbiota transplantation (FMT) provides the strongest proof-of-concept for re-sensitization in anti–PD-1–refractory melanoma. Microbiome repair can also improve refractory ICI-associated colitis. Early trials of live biotherapeutics and defined consortia support scalability but highlight context dependence and design pitfalls, including antibiotic preconditioning. We discuss practical determinants of reproducibility, including co-medications, diet, engraftment and functional readouts, and conclude with safety, regulatory, and reporting priorities for clinically deployable microbiome engineering.

## 1 Introduction

Immune checkpoint inhibitors (ICIs) have transformed the management of multiple malignancies, yet durable benefit remains restricted to a subset of patients, and immune-related adverse events (irAEs) can limit treatment intensity and continuity (1–3). Tissue-based biomarkers such as PD-L1 expression, microsatellite instability, and tumor mutational burden have advanced patient selection, but they do not fully capture host-level determinants that shape response and toxicity trajectories (4). One increasingly compelling host factor is the gut microbiome, which has moved from a correlative “omics” signal to a biologically grounded and clinically testable variable (5).

Accumulating evidence indicates that gut microbial features associate with ICI outcomes across tumor types (6). For example, enrichment of Ruminococcaceae has been linked to response in melanoma and liver cancer, while *Akkermansia muciniphila* has repeatedly been observed in responders across epithelial tumors, including lung and renal cell carcinomas (6–8). These observations have accelerated mechanistic work and, importantly, have begun to motivate intervention studies that aim not only to predict ICI benefit but to actively enhance it through microbiome-targeting strategies (9, 10). Such strategies are diverse, spanning dietary optimization and prebiotics, precision probiotics and defined bacterial consortia, fecal microbiota transplantation (FMT), and, in selected contexts, approaches that reshape ecological pressures such as antibiotic stewardship or bacteriophage-based modulation (11–15).

**Figure 1:**
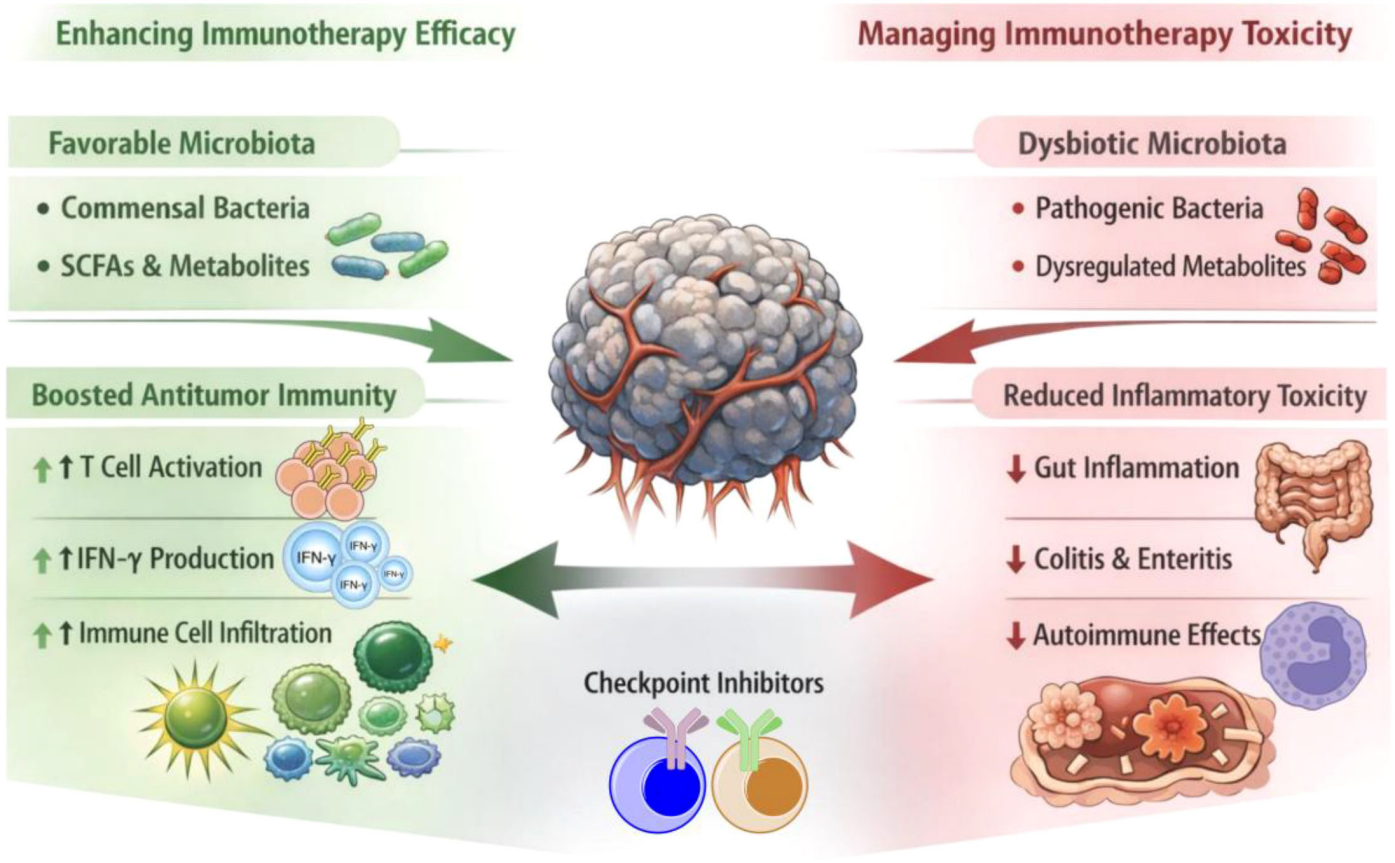
Conceptual framework for microbiome engineering to enhance checkpoint immunotherapy efficacy and manage immune-related toxicity. Favorable microbiota and their metabolites support antitumor immunity by promoting T cell activation, IFN-γ–associated effector programs, and immune cell infiltration into tumors, thereby increasing the likelihood of benefit from checkpoint inhibitors. In parallel, microbiome states associated with dysbiosis can shape inflammatory tone at barrier sites and influence the risk of immune-related adverse events, including gastrointestinal inflammation such as colitis and enteritis. The schematic highlights the dual opportunity of microbiome modulation to broaden the therapeutic window of anti–PD-1 and anti–CTLA-4 therapies through coordinated effects on efficacy and tolerability.

A key premise underlying these efforts is that microbiome engineering may help broaden the therapeutic window of checkpoint blockade by jointly shaping antitumor efficacy and inflammatory toxicity (16). As summarized in **Figure 1**, favorable microbiota states and their metabolites can support T cell activation, IFN-γ–associated effector programs, and immune cell infiltration, thereby increasing the likelihood of benefit from anti–PD-1 and anti–CTLA-4 therapy. Conversely, microbiome states consistent with dysbiosis may contribute to heightened inflammatory tone at epithelial barrier sites and may be associated with clinically consequential irAEs, particularly gastrointestinal inflammation such as colitis and enteritis (17). This dual-impact framework also helps explain why the same intervention may have divergent outcomes across patients, depending on baseline ecology, co-medications, and the immune set point. This mini-review provides an intervention-oriented synthesis of the field. We summarize biological principles linking gut microbes and their products to ICI pharmacology, review clinical evidence for three leading intervention modalities—FMT, live biotherapeutic products, and defined consortia—and discuss translational factors that govern reproducibility, safety, and clinical integration.

## 2 Biological basis for microbiome effects on ICI

### 2.1 Antigen presentation and T cell priming

A central mechanistic theme is that gut microbes can tune the quality of antitumor T cell responses by shaping antigen presentation and the balance between effector and regulatory programs. In preclinical models, specific commensals promote dendritic cell activation and cross-priming, resulting in more robust CD8^+^ T cell expansion and improved tumor control under checkpoint blockade (18, 19). In humans, this concept is indirectly supported by interventional studies in which FMT from ICI responders into anti–PD-1–refractory melanoma recipients was accompanied by immune remodeling consistent with enhanced antitumor immunity, including changes in intratumoral immune composition and inflammatory programs (20). Although these early trials were not designed to prove a single dominant pathway, they provide clinical plausibility that microbiome manipulation can shift the host immune set point in a direction that is permissive for renewed ICI activity.

A related and clinically relevant layer is that microbiome–immune interactions may influence not only the magnitude of T cell responses but also their spatial deployment within tumors. When the microbiome supports systemic immune readiness, tumor microenvironments that are otherwise “immune-excluded” may become more accessible to cytotoxic effectors once PD-1/PD-L1 signaling is blocked (21). This framing helps explain why microbiome interventions have been explored primarily in refractory disease settings, where overcoming a pre-existing barrier to effective T cell function is a pragmatic therapeutic goal.

### 2.2 Metabolite–immune coupling

Beyond taxonomic composition, the functional output of the microbiome is increasingly viewed as a key driver of ICI sensitivity. Microbial metabolites and products can influence T cell fitness, myeloid polarization, and inflammatory tone through pathways that are, in principle, measurable and modifiable (22, 23). A clinically tangible example is dietary fiber, which alters short-chain fatty acids (SCFAs) production and broader metabolic ecology. In a cohort of melanoma patients receiving immune checkpoint blockade, higher dietary fiber intake was associated with improved progression-free survival, whereas over-the-counter probiotic use correlated with worse outcomes, highlighting that commonly used “health” interventions can materially shift the host–microbiome–immunity axis during ICI therapy (11). These observations do not establish causality on their own, but they align with a function-first view in which metabolites and ecological stability, rather than single taxa, may determine whether the immune system can sustain an effective response under checkpoint blockade.

Beyond taxonomy-level associations, microbial metabolites may provide a more functionally interpretable link between ecosystem composition and ICI responsiveness. In particular, short-chain fatty acids such as butyrate can reshape innate immune programming and mucosal inflammatory tone, supporting the concept that metabolite-level outputs may influence downstream antitumor immunity. More broadly, current ICI-focused microbiome literature also supports the view that functional microbial products, rather than taxonomy alone, may serve as a more transferable layer for mechanistic interpretation and future biomarker development (24, 25).

### 2.3 Barrier integrity, systemic inflammation, and links to irAEs

Microbiome effects on ICI are bidirectional because the same host–microbe interface that shapes antitumor immunity also influences epithelial barrier function and systemic inflammation, which are central to immune-related toxicity. Clinically, irAEs often involve barrier organs such as the gut and skin, and emerging patient cohorts suggest that baseline gut microbiome features may associate with subsequent development of irAEs during ICI (17). This observation is important for microbiome engineering because it argues that the field should not optimize for antitumor activity alone; instead, interventions should aim to widen the therapeutic window by supporting antitumor immunity while avoiding ecological states that predispose to severe inflammatory toxicity.

Real-world medication exposures further underscore how fragile this balance can be. Antibiotics given shortly before or after ICI initiation have repeatedly been associated with worse outcomes across observational studies and meta-analyses, supporting the idea that abrupt ecological disruption can impair beneficial immune conditioning (11). This does not mean antibiotics should be avoided when clinically indicated, but it motivates stewardship and careful trial design when microbiome modulation is combined with ICIs. Together, these data support a working model in which microbiome composition and function influence both efficacy and toxicity via immune priming, metabolite signaling, and barrier-associated inflammatory set points, providing multiple mechanistic entry points for intervention.

## 3 Clinical evidence for microbiome intervention strategies

Clinical interest in microbiome engineering has progressed from retrospective associations to prospective intervention studies that directly test whether reshaping the gut ecosystem can alter the clinical course of checkpoint immunotherapy (26–28). Across modalities, three recurring themes have emerged. First, microbiome interventions appear most informative in settings where the unmet need is largest, particularly anti–PD-1–refractory disease, because even modest re-sensitization signals are clinically meaningful. Second, clinical activity is consistently accompanied by evidence of ecological change in the gut, yet engraftment is not uniformly sufficient to guarantee response, implying that tumor-intrinsic factors and the pre-existing immune set point constrain the ceiling of benefit. Third, the clinical value of microbiome engineering is likely to depend on standardization and patient stratification, because baseline microbiome states, prior antibiotics, diet, and co-medications can materially shape the effectiveness of a given intervention (29, 30).

### 3.1 Fecal microbiota transplantation

Fecal microbiota transplantation has provided the strongest early proof-of-concept that microbiome manipulation can modulate checkpoint outcomes in humans. Two independent phase 1 studies in anti–PD-1–refractory metastatic melanoma adopted a similar clinical logic, namely transferring fecal communities from donors who had benefited from anti–PD-1 therapy and then re-challenging recipients with PD-1 blockade. In the trial reported by Davar and colleagues, a subset of refractory patients achieved objective responses or durable disease control, together with donor-like microbiome features and tumor immune remodeling consistent with a more permissive anti-tumor immune state (31). In parallel, Baruch and colleagues observed clinical responses after responder-derived FMT followed by anti–PD-1 reinduction, again coupled to changes in gut community structure and immune correlates (20). Although these studies were small and not powered to define predictors of benefit, their convergence is important because it supports a causal interpretation that microbiome state can contribute to therapeutic sensitivity rather than simply reflecting it (32).

Equally valuable are the practical lessons these trials offer for next-generation designs. Donor selection is a plausible determinant of effect size, but even with “responder donors,” not all recipients respond, suggesting that microbiome transfer interacts with tumor and host constraints (33, 34). Moreover, the need to achieve and maintain meaningful engraftment highlights an often underappreciated issue in oncology trials, namely that microbiome interventions behave less like a single agent and more like an ecological perturbation whose durability depends on the recipient environment. Although the clearest proof-of-concept remains in anti–PD-1–refractory melanoma, emerging prospective studies have begun to evaluate FMT-based strategies in first-line settings and in additional tumor types, including melanoma, NSCLC, and metastatic renal cell carcinoma (9, 10, 35). These considerations have motivated broader exploration beyond melanoma and the development of more standardized products (36).

### 3.2 Live biotherapeutic products

Live biotherapeutic products aim to deliver microbiome modulation with tighter manufacturing control and scalability than FMT. A widely discussed clinical example is CBM588 (a live bacterial preparation) tested in a randomized phase 1 setting in metastatic renal cell carcinoma with a standard checkpoint backbone. In this study, nivolumab plus ipilimumab administered with CBM588 showed signals consistent with improved clinical outcomes compared with checkpoint blockade alone, without an obvious increase in toxicity, while also demonstrating that supplementation can measurably reshape stool microbiome features in patients receiving combination ICI (12). The broader implication is not that any single strain is universally beneficial, but that a defined live product can, under some conditions, be integrated into routine ICI regimens and yield clinically relevant signals.

At the same time, results across live biotherapeutic programs have been variable, and this variability is itself informative for the field (37, 38). Compared with FMT, which transfers a complex community with multiple functional modules, Live biotherapeutic products (LBPs) may be more sensitive to baseline ecology, substrate availability, and co-medications that influence colonization and metabolite output. These dependencies suggest that future trials may need to prioritize function-based stratification and pharmacodynamic readouts (for example, engraftment metrics and metabolomic signatures) rather than relying primarily on taxonomic endpoints.

### 3.3 Defined consortia

Defined consortia represent an intermediate approach between FMT and single-product supplementation, seeking to combine standardization with ecological breadth. Early consortia programs in melanoma illustrate both promise and pitfalls. In a randomized, placebo-controlled, biomarker-stratified phase Ib study evaluating the spore-based consortium SER-401 with nivolumab, the trial was ultimately underpowered because of low accrual, limiting efficacy conclusions; however, the translational findings provided a concrete warning regarding broad antibiotic preconditioning (39). Specifically, vancomycin preconditioning substantially disrupted the microbiome and was associated with impaired systemic immunity with incomplete recovery at the time of ICI initiation, raising the possibility that an overly aggressive “reset” can remove beneficial functions needed for effective checkpoint priming. This type of negative or mixed outcome is highly instructive because it reframes trial design away from simple “deplete then add” logic and toward more context-aware strategies that preserve key functions while adding missing modules.

Taken together, the current interventional landscape supports a pragmatic interpretation. FMT provides the clearest human proof-of-concept for re-sensitization in refractory settings and for immune remodeling that tracks with benefit. Live biotherapeutics and defined consortia offer routes to standardization and scalability, but their clinical impact may depend more strongly on baseline community states, co-medications, and the ability to achieve durable function in the recipient. These insights set the stage for the next translational question addressed in subsequent sections, namely how to improve reproducibility and safety while aligning microbiome interventions with clinically actionable endpoints. Representative clinical studies are summarized in **Table 1**, highlighting modality-specific signals, pharmacodynamic readouts, and recurrent design constraints.

**Table 1:** Representative clinical trials and published studies of microbiome interventions used to enhance ICI efficacy or manage ICI-related toxicity.

Study/Trial	Intervention	Cancer type/setting	Design/ICI backbone	Key findings	Ref.
Davar et al., 2021	FMT (efficacy)	Metastatic melanoma, anti–PD-1 refractory	Phase 1; responder-donor FMT followed by anti–PD-1 rechallenge	A subset achieved renewed clinical benefit; donor-like engraftment and tumor/systemic immune remodeling were observed. Small sample size and heterogeneous prior therapy limit generalizability.	(31)
Baruch et al., 2021	FMT (efficacy)	Metastatic melanoma, immunotherapy refractory	Phase 1; responder-donor FMT with anti–PD-1 reinduction	Clinical responses were seen in a subset, with accompanying ecological shift and immune correlates. Durability was variable, and baseline ecological context/co-medications may influence outcomes.	(20)
Dizman et al., 2022	Live biotherapeutic product (CBM588)	Metastatic renal cell carcinoma, first-line ICI combination setting	Randomized phase 1; nivolumab + ipilimumab	Feasibility was demonstrated, with exploratory signals consistent with improved outcomes. Stool metagenomic changes supported microbiome modulation, but the study was early-phase and underpowered.	(12)
SER-401 phase Ib (Glitza et al., 2024)	Defined consortium (SER-401)	Advanced melanoma, ICI-treated population	Randomized, placebo-controlled, biomarker-stratified phase Ib; nivolumab; included vancomycin preconditioning	The trial was underpowered for efficacy, but provided an important design lesson: antibiotic preconditioning caused major microbiome disruption and incomplete immune recovery at ICI initiation.	(39)
Case series	FMT (irAE rescue)	ICI-associated colitis refractory to standard management	Case series; no fixed ICI backbone applicable	Clinical improvement was reported in refractory colitis, with microbiome reconstitution and mucosal immune changes suggestive of restored tolerance. Small numbers and infection risk require rigorous screening/monitoring.	(40)
Translational clinical study	FMT (irAE rescue)	Refractory ICI-induced colitis	Translational clinical study; no fixed ICI backbone applicable	FMT showed activity in refractory cases, supporting ecological repair as a therapeutic option. Not randomized; optimal timing relative to immunosuppressants remains incompletely defined.	(41)

## 4 Translational considerations for reproducible clinical benefit

A major translational challenge in microbiome engineering is that the intervention is not delivered into a neutral background. Rather, it enters a dynamic ecological and clinical context shaped by tumor type, prior therapies, diet, geography, and supportive medications. In practice, two patients receiving the same microbiome product can experience very different functional exposures, because baseline community structure constrains colonization, metabolite output, and immune coupling. This is one reason why the field is moving from taxonomy-only descriptions toward biomarker frameworks that emphasize dynamic, patient-specific readouts and clinical implementability (42, 43).

A first design principle is to treat baseline context and co-medications as core trial variables rather than nuisance covariates. Antibiotic exposure is the most consistent example. Multiple systematic reviews and meta-analyses have associated antibiotics given shortly before or around ICI initiation with worse outcomes across cancers, and more recent analyses continue to support this concern in contemporary immunotherapy-treated populations (44, 45). In microbiome-intervention trials, this issue becomes even more concrete. In the biomarker-stratified SER-401 trial, translational analyses suggested that vancomycin preconditioning disrupted the gut microbiota and coincided with impaired systemic immunity with incomplete recovery at the time of ICI initiation, cautioning that “deplete then add” strategies can unintentionally remove beneficial functions needed for effective checkpoint priming (39). These observations do not argue against clinically indicated antibiotics; they argue for stewardship, careful timing documentation, and preplanned subgroup analyses.

A second principle is to plan for ecological pharmacology and verify that the intended biological exposure is achieved. FMT studies in anti–PD-1–refractory melanoma demonstrated that donor-like shifts and sustained engraftment are measurable in humans and often accompany immune remodeling, but engraftment alone does not guarantee clinical response (46). For LBPs and defined consortia, engraftment may be even more sensitive to the recipient environment, making it important to prospectively define pharmacodynamic readouts. Depending on feasibility, these can include strain-level metagenomics for colonization, metabolomic signatures (for example, SCFAs- or bile-acid–related pathways), and peripheral immune metrics aligned to the intervention hypothesis. The practical goal is not to measure everything, but to ensure that a negative trial can distinguish “product failure” from “delivery failure.”

A third consideration is the interaction between microbiome engineering and common supportive medications that can shape inflammation and microbial ecology. Baseline systemic corticosteroid use at the start of PD-(L)1 blockade has been associated with poorer outcomes in advanced NSCLC, reinforcing the broader point that immunosuppressive tone at ICI initiation can constrain response and may confound microbiome-intervention effects (47). Similarly, proton pump inhibitor exposure has been associated with reduced survival in ICI-treated populations in recent systematic reviews and meta-analyses, consistent with the possibility that acid suppression perturbs the gut ecosystem in ways that are unfavorable for checkpoint efficacy in some contexts (48). Dietary behavior is another modifiable co-variable. In melanoma cohorts, higher dietary fiber intake was associated with improved outcomes on ICI, whereas commercially available probiotic supplement use correlated with less favorable outcomes in some analyses; these clinical observations, supported by parallel mechanistic experiments, underscore that “microbiome engineering” is partly an ecological management problem that may require attention to substrates and competing exposures (11).

Finally, reproducibility depends on choosing endpoints and trial structures that match the intended claim. In refractory disease, small early-phase studies can reasonably focus on objective response or durable disease control coupled with predefined microbiome and immune pharmacodynamics, because even modest re-sensitization signals are informative. In frontline settings, where background response rates can be higher, randomized designs and prespecified subgroups may be necessary to avoid false positives and to identify the patients most likely to benefit. Across settings, trials are strengthened when they include a minimal set of standardized reporting elements, including timing of antibiotics, steroids, and acid suppressants, baseline microbiome features, engraftment or functional readouts, and safety monitoring plans (49).

## 5 Safety, regulatory issues, and reporting standards

Safety is the primary gating factor for microbiome engineering in immunotherapy because the “active ingredient” is a living ecosystem administered to patients who may be immunocompromised, heavily pretreated, or concurrently receiving immunomodulators (50, 51). For FMT, the dominant hazards are transmissible infections and the imperfect detectability of donor-borne pathogens at the time of donation, as illustrated by documented transmission of drug-resistant *Escherichia coli* bacteremia following investigational FMT, including a fatal outcome (52). Subsequent safety concerns have extended beyond classical multidrug-resistant organisms to include enteric pathogens such as enteropathogenic *E. coli* and Shiga toxin–producing *E. coli*, reinforcing that screening must evolve with both “old bugs” and newly recognized threats (53). During the COVID-19 pandemic, expert guidance emphasized the plausibility of SARS-CoV-2 transmission via donor stool and recommended urgent updates to donor screening and investigational-use safeguards (54). Together, these events translate into a practical implication for oncology-oriented programs: rigorous donor screening, traceability, controlled manufacturing, and clear stopping rules are prerequisites for ethically defensible trials rather than optional add-ons.

Regulatory frameworks shape feasibility and scalability because most oncology-relevant microbiome interventions remain investigational and are expected to meet biologic drug standards for identity, purity, potency-related controls, and lot-to-lot consistency, particularly for live biotherapeutic products (LBPs) (55). Recent approvals of microbiota-based therapeutics for recurrent *Clostridioides difficile* infection provide an instructive precedent for how donor-sourced or purified-consortium products can be developed within regulated pathways with product-specific manufacturing controls and safety follow-up, even though these are not oncology indications (56, 57). Reproducibility also depends on reporting standards that match the complexity of microbiome interventions; conventional oncology reporting often omits decisive details such as stool collection and storage logistics, sequencing and bioinformatics pipelines, and contemporaneous exposures (antibiotics, proton pump inhibitors, diet, probiotics) that can confound interpretation (49). For microbiome–ICI studies, a minimal reporting core should include donor or product screening procedures when applicable, prespecified engraftment or functional pharmacodynamic endpoints, adverse-event attribution frameworks, and transparent handling of missing data, so that negative trials can distinguish “product failure” from “delivery failure” and enable credible cross-study synthesis.

## 6 Conclusions and future directions

The gut microbiome has progressed from a correlational biomarker domain to an intervention space with tangible clinical signals in checkpoint immunotherapy. Across studies, the most consistent message is that microbiome state can contribute to both the probability of antitumor benefit and the risk profile of immune-related toxicity, positioning microbiome engineering as a strategy to improve the therapeutic window rather than simply to “predict responders.” The strongest human proof-of-concept for efficacy modulation comes from responder-derived FMT in anti–PD-1–refractory melanoma, where a subset of patients achieved renewed clinical benefit together with measurable ecological shifts and immune remodeling. Standardized approaches are beginning to emerge through live biotherapeutic products, exemplified by early randomized experience combining CBM588 with nivolumab–ipilimumab in metastatic renal cell carcinoma, although larger and more definitive trials will be needed to determine generalizability and identify the patients most likely to benefit. At the same time, mixed or underpowered consortia programs have offered valuable design lessons, including the possibility that broad antibiotic preconditioning may inadvertently disrupt protective immune readiness and compromise subsequent checkpoint activity.

A near-term priority is to improve reproducibility by treating microbiome interventions as “ecological pharmacology” rather than as simple add-ons. This implies prespecified, feasible pharmacodynamic readouts that confirm biological exposure, such as engraftment metrics and functional signatures, alongside systematic capture of confounders including antibiotics, diet, corticosteroids, and acid suppressants. Equally important is safety-forward development. Severe infectious complications after investigational FMT, including transmission of drug-resistant organisms, underscore the need for rigorous screening, traceability, and controlled manufacturing, especially in immunocompromised populations. On the toxicity-management side, FMT has demonstrated promising activity in refractory immune-mediated colitis, supporting the broader concept that targeted ecosystem repair can restore mucosal immune balance when standard immunosuppression is insufficient. Looking ahead, progress is most likely to come from function-first stratification, standardized reporting, and trial designs aligned to clinically meaningful claims. Rather than searching for universally “good” taxa, future programs will likely focus on transferable functions and measurable outputs that can be integrated with established biomarker frameworks for checkpoint therapy.
